# Chained structure of directed graphs with applications to social and transportation networks

**DOI:** 10.1007/s41109-022-00502-x

**Published:** 2022-09-05

**Authors:** Anna Concas, Caterina Fenu, Lothar Reichel, Giuseppe Rodriguez, Yunzi Zhang

**Affiliations:** 1 Dipartimento di Matematica e Informatica, Università di Cagliari, Via Ospedale 72, 09124 Cagliari, Italy; 2 Department of Mathematical Sciences, Kent State University, Kent, 44242 OH USA; 3 Department of Data Science and Statistics, Dongbei University of Finance and Economics, Dalian, 116025 China

**Keywords:** Network analysis, Directed chained graph, Central vertex, 05C50, 05C82, 91D30

## Abstract

The need to determine the structure of a graph arises in many applications. This paper studies directed graphs and defines the notions of $\ell$-chained and $\{\ell ,k\}$-chained directed graphs. These notions reveal structural properties of directed graphs that shed light on how the nodes of the graph are connected. Applications include city planning, information transmission, and disease propagation. We also discuss the notion of in-center and out-center vertices of a directed graph, which are vertices at the center of the graph. Computed examples provide illustrations, among which is the investigation of a bus network for a city.

## Introduction

A complex system that is composed of separate items that are interconnected in some way can be modeled by a network. Networks are represented by graphs, which are made up of nodes and edges. The latter connect the nodes. Networks arise in many areas of science and engineering, such as biology, communication, transportation, and social media; see e.g., Estrada (2012), Newman (2010) for discussions of these and many other applications.

The edges in a network may have weights, which are real values and generally positive, and may measure the strength of the interaction between linked nodes. The connections may have a direction. A graph is referred to as *undirected* if all edges are undirected, i.e., they are “two-way streets;” a graph with at least one directed edge (which can be thought of as a “one-way street”) is said to be *directed*. We are concerned with *directed* *unweighted* graphs without self-loops. Thus, all edges have the same weight (which we set to one), and there are no edges from a node back to itself.

A considerable number of mathematical and computational methods for studying networks have been developed. Among the aims of network analysis is the identification of the most important nodes or edges of a graph by using the notion of centrality, which first arose in the context of social science, or to determine the structure of the underlying graph; see, e.g., De la Cruz Cabrera et al. (2020), De la Cruz Cabrera et al. (2021), Estrada (2012), Estrada and Higham (2010), Newman (2010) for many examples.

A fundamental topological property of a graph, which will be briefly recalled in the section “Notation and some properties of graphs and networks”, is *multipartivity*. The nodes in an *m*-partite graph can be split into *m* disjoint subsets ${{\mathcal {V}}}_i$, $i=1,2,\ldots ,\ell$, called *partite sets*, with connections occurring only between the subsets, but not within the subsets. When $\ell=2$, the graph is said to be *bipartite*. A refinement of bipartivity for undirected graphs, referred to as the *chained structure* of the graph, was introduced in Concas et al. (2021). The chained structure characterizes undirected multipartite graphs; an *m*-chained graph has only edges between nodes that belong to “subsequent” partite sets ${{\mathcal {V}}}_i$ and ${{\mathcal {V}}}_{i+1}$, $i = 1, 2, \ldots , \ell-1$ (and vice versa). This paper extends the notion of chained graphs from undirected graphs, to directed graphs. The chained structure reveals the “depth” of a graph, i.e., how many steps it may take to go from a specified node to any other node, by following edges along their direction.

In Concas et al. (2021), we used chained graphs to identify *central nodes* by introducing the position centrality measure for nodes of an undirected graphs. This notion is a generalization of closeness centrality. Central nodes are identified by their location in the chained structure. For an overview of other centrality measures; see Borgatti (2005), Estrada and Higham (2010), Estrada and Rodriguez-Velazquez (2005). This paper generalizes position centrality to directed graphs. Specifically, for directed graphs that have directed spanning trees, we define *in-position* and *out-position* centralities of a node by examining two different types of directed spanning trees associated with the graph; see Gabow and Myers (1978) for a discussion on directed spanning trees. These centrality concepts shed light on the ease of communication within a network. In the two sections devoted to numerical examples, we compare them to other existing centrality measures. In general, it is impossible to state which centrality measure is the best, as the concept of centrality takes different meanings in different applications. What we show is that position centrality, by varying the value of the parameter on which it depends, is able to spot specific aspects of a network that are not detected by traditional measures, and that depend upon the underlying chained structure.

The identification of the chained structure of a directed graph also can be useful for detecting the presence of anti-communities, i.e., node subsets that are loosely connected internally, but have many external connections with the rest of the graph. Several methods have been developed to identify anti-communities in undirected graphs; see Concas et al. (2020), Estrada and Knight (2015), Fasino and Tudisco (2017). The relation between clustering and community detection in directed graphs has been discussed in Laenen and Sun (2020). In Concas et al. (2021), we illustrated how the chained structure may be used for introducing a density measure for computing an “anti-community score” for undirected graphs. We extend this measure to directed graphs in the present paper. To the best of our knowledge, while the identification of anti-communities has been studied in the literature (Estrada and Knight 2015) (see also Concas et al. 2020; Fasino and Tudisco 2017), the identification of near-anti-communities (which are associated with a small anti-community score) has not been discussed yet.

This paper is organized as follows. The section “Notation and some properties of graphs and networks” introduces notation and discusses general properties of graphs that will be used later. Directed chained graphs are defined in the section “[Sec Sec3]”. They can be studied with the aid of directed spanning trees. This is discussed in the section “Directed chained graphs and directed spanning trees”. The chained structure naturally leads to the concept of position centrality, defined in “Position centrality and some applications” section. Nodes with the largest position centrality are referred to as central nodes. Some data sets deriving from real-world applications, including a social network, are analyzed in the section “Some examples”. The  section “A case study about position centrality” sheds light on the properties of center nodes by considering a case study concerning a bus transportation network. Finally, the section “Conclusion” contains concluding remarks.

## Notation and some properties of graphs and networks

A network can be represented by a graph ${{\mathcal {G}}}=\{{{\mathcal {V}}},{{\mathcal {E}}}\}$, where ${{\mathcal {V}}}=\lbrace v_i\rbrace _{i=1}^n$ is a set of *nodes*, or *vertices*, and ${{\mathcal {E}}}=\lbrace e_i\rbrace _{i=1}^m$ a set of *edges*, which connect the nodes. Two nodes $v_i$ and $v_j$, for $i\ne j$, are said to be *adjacent* if there is an edge from node $v_i$ to node $v_j$. In this context, an undirected edge between the nodes $v_i$ and $v_j$ points both from $v_i$ to $v_j$ and from $v_j$ to $v_i$. The node $v_i$ is said to be *connected* to the node $v_j$ if there is a path from $v_i$ to $v_j$, that is, if there is a sequence of edges $\{e_{r_s}\}_{s=1}^k$ such that $e_{r_1}$ originates from $v_i$, $e_{r_k}$ points to $v_j$, and if $e_{r_s}$ points to $v_\ell$, then $e_{r_{s+1}}$ starts from the same node for $s=1,2,\ldots ,k-1$. A cycle is a path that starts and ends at the same node $v_i$.

An undirected graph is *connected* if each pair of distinct nodes is connected by a path. A directed graph is said to be *strongly connected* if for each vertex pair $(v_i,v_j)$ the node $v_i$ is connected to the node $v_j$, and the node $v_j$ is connected to $v_i$. A directed graph is said to be *semi-connected* if for each vertex pair $(v_i,v_j)$ either the vertex $v_i$ is connected to the vertex $v_j$, or $v_j$ is connected to $v_i$. A directed graph is *weakly connected* if there is a path between each vertex pair $(v_i,v_j)$ in the underlying undirected graph, that is, in the undirected graph obtained by replacing all directed edges by undirected ones. We refer to Estrada (2012) and Newman (2010) for discussions on graphs and their properties.

An unweighted graph ${{\mathcal {G}}}$ with *n* vertices can be represented by an adjacency matrix $A=[a_{ij}]_{i,j=1}^n$ with $a_{ij}=1$ if there is an edge from vertex $v_i$ to vertex $v_j$; otherwise $a_{ij}=0$. Since an undirected edge can be thought of as being made up of two directed edges (in opposite directions), the adjacency matrix of an undirected graph is symmetric; the adjacency matrix of a directed graph is nonsymmetric.

Multipartivity and, in particular, bipartivity are fundamental topological characteristics of graphs that model interactions between different types of objects. Bipartite graphs contain vertices that can be partitioned into two disjoint vertex subsets ${{\mathcal {V}}}_1$ and ${{\mathcal {V}}}_2$, such that there are no connections between vertices in the same subset. Assume that the *n* vertices of a bipartite graph ${{\mathcal {G}}}$ are separated so that the first $n_1$ vertices make up the vertex set ${{\mathcal {V}}}_1$ and the remaining $n_2=n-n_1$ vertices make up the vertex set ${{\mathcal {V}}}_2$. Then the adjacency matrix *A* of ${{\mathcal {G}}}$ is of the form

2.1 $$\begin{aligned} A=\begin{bmatrix} O & C_1\\ C_2 & O \end{bmatrix}, \end{aligned}$$

where $C_1\in {\mathbb {R}}^{n_1\times n_2}$, $C_2\in {\mathbb {R}}^{n_2\times n_1}$, and *O* denotes a zero-matrix of suitable order. If the graph ${{\mathcal {G}}}$ is undirected, then $C_2=C_1^T$, where the superscript $^T$ denotes transposition.

In undirected $\ell$-chained graphs, the nodes are divided into $\ell$ disjoint subsets

2.2 $$\begin{aligned} {{\mathcal {V}}}={{\mathcal {V}}}_1\cup {{\mathcal {V}}}_2\cup \cdots \cup {{\mathcal {V}}}_{\ell } \end{aligned}$$

so that there are edges only between nodes belonging to “adjacent” node sets, that is, all edges from a node in ${{\mathcal {V}}}_i$ point to a node in ${{\mathcal {V}}}_{i+1}$ or in ${{\mathcal {V}}}_{i-1}$ for some *i*. This kind of partitioning is discussed in Concas et al. (2021).

## Directed $\ell$-chained graphs and their adjacency matrices

The directed chained graphs introduced in this section generalize the notion of undirected chained graphs defined in Concas et al. (2021).

### Definition 1

A directed graph ${{\mathcal {G}}}= \{{{\mathcal {V}}},{{\mathcal {E}}}\}$ is said to be directed $\ell$-*chained*, with initial vertex $v_i$, if the set of vertices can be subdivided into $\ell$ disjoint non-empty subsets ${{\mathcal {V}}}_1,{{\mathcal {V}}}_2,\ldots ,{{\mathcal {V}}}_\ell$, see (2.2), such that $v_i\in {{\mathcal {V}}}_1$ and all edges from vertices in the set ${{\mathcal {V}}}_j$ point to vertices in the set ${{\mathcal {V}}}_{j+1}$ for $j=1,2,\ldots ,\ell -1$, where the chain length $\ell$ is the largest number of vertex subsets ${{\mathcal {V}}}_j$ with this property. The vertex subset ${{\mathcal {V}}}_{j+1}$ is said to be adjacent to the vertex set ${{\mathcal {V}}}_j$.

The chain length $\ell$ of a directed $\ell$-chained graph may depend on the choice of the initial vertex $v_i$. After a suitable permutation of the nodes, the adjacency matrix *A* of a directed $\ell$-chained graph ${{\mathcal {G}}}= \{{{\mathcal {V}}},{{\mathcal {E}}}\}$ becomes upper block bidiagonal with zero diagonal blocks,

3.1 $$\begin{aligned} A = \begin{bmatrix} O & A_1 \\ & O & A_2 \\ & & O & A_3 \\ & & & \ddots & \ddots \\ & & & & O & A_{\ell -1} \\ & & & & & O \end{bmatrix}, \end{aligned}$$

where the submatrix $A_i\in {{\mathbb {R}}}^{n_i\times n_{i+1}}$ describes the connections from vertices in ${{\mathcal {V}}}_i$ to vertices in ${{\mathcal {V}}}_{i+1}$, for $i=1,2,\ldots ,\ell -1$.

**Fig. 1:**
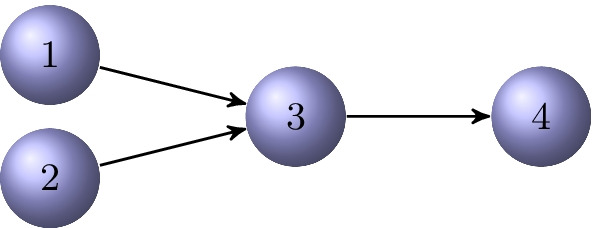
A directed 3-chained graph ${{\mathcal {G}}}$ with initial vertex $v_1$

### Example 3.1

Consider the graph of Fig. 1. This is a 3-chained graph with the chained node sets ${{\mathcal {V}}}_1=\{v_1,v_2\}$, ${{\mathcal {V}}}_2=\{v_3\}$, and ${{\mathcal {V}}}_3=\{v_4\}$. The initial node can be chosen to be either $v_1$ or $v_2$. The adjacency matrix is

$$\begin{aligned} A=\left[ \begin{array}{cccc} 0 & 0 & 1 & 0 \\ 0 & 0 & 1 & 0 \\ 0 & 0 & 0 & 1 \\ 0 & 0 & 0 & 0 \end{array}\right] , \end{aligned}$$

where we can choose the submatrices

$$\begin{aligned} A_1=\left[ \begin{array}{c} 1 \\ 1 \end{array}\right] \in {{\mathbb {R}}}^{2\times 1},\qquad A_2=\left[ \begin{array}{c} 1 \end{array}\right] \in {{\mathbb {R}}}^{1\times 1}. \end{aligned}$$

Assume that a graph is known to be directed $\ell$-chained for some $\ell \ge 1$, but that the value of $\ell$ is not known. Moreover, let a permuted version of the matrix (3.1) be known (for some unknown value of $\ell$). Thus, the available adjacency matrix is of the form

$$\begin{aligned} {\widetilde{A}} = P A P^T, \end{aligned}$$

where *P* is a permutation matrix that modifies the vertex ordering. Given the adjacency matrix ${\widetilde{A}}$, we are interested in determining the vertex subsets ${{\mathcal {V}}}_1,{{\mathcal {V}}}_2,\ldots ,{{\mathcal {V}}}_\ell$ in Definition 1, as well as the number of sets $\ell \ge 1$. A method for determining if a directed graph is $\ell$-chained and partitioning the nodes into subsets is described by Algorithm 1. Given an adjacency matrix *A* of a directed graph, the first node subset ${{\mathcal {V}}}_1$ is obtained by considering the column indices *j* such that $A_{ij}=0$ for each row index *i*; see line 1 of the algorithm. Then the other vertex subsets are determined by identifying the blocks in *A* that describe connections with nodes in the preceding node subset (line 6). If it is not possible to determine the first vertex set, or if during the process it results that some node is connected to a vertex in a preceding subset, then the graph is not $\ell$-chained. This process gives a constructive proof of the following result.

### Proposition 1

Let ${{\mathcal {G}}}=\{{{\mathcal {V}}},{{\mathcal {E}}}\}$ be a directed graph. Then it is possible to detect if it possesses an $\ell$-chained structure and determine the number of subsets, $\ell$, as well as the vertex set partitioning ${{\mathcal {V}}}={{\mathcal {V}}}_1\cup {{\mathcal {V}}}_2\cup \cdots \cup {{\mathcal {V}}}_\ell$.


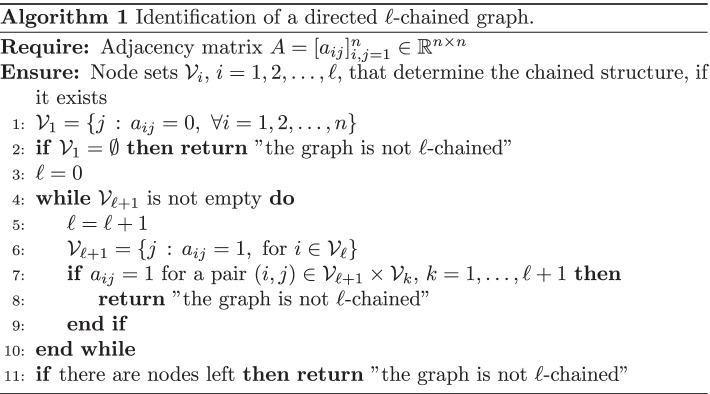


The definition of directed $\ell$-chained graphs is quite restrictive. To be able to discuss properties of a larger set of directed graphs, we relax the requirements of Definition 1 to allow edges between vertices in the vertex subset ${{\mathcal {V}}}_i$ to vertices in vertex subset ${{\mathcal {V}}}_j$ for some $j\le i$ with *j* not much smaller than *i*.

### Definition 2

The directed graph ${{\mathcal {G}}}= \{{{\mathcal {V}}},{{\mathcal {E}}}\}$ is said to be directed $\{\ell ,k_i\}$-*chained* with initial vertex $v_i$ if it has the chained structure described in Definition 1 with the extension that edges from vertices in the set ${{\mathcal {V}}}_j$ are allowed to point to vertices in the sets ${{\mathcal {V}}}_{\max \{j-k_i,1\}},\ldots ,{{\mathcal {V}}}_j,{{\mathcal {V}}}_{j+1}$ for $j=1,2,\ldots ,\ell -1$ and some $k_i\ge 0$. The integer $k_i$, which we refer to as the *lower bandwidth*, is the largest integer with this property.

We note that Definition 1 corresponds to the situation when $k_i=-1$ for all *i* in Definition 2.

### Definition 3

The minimal lower bandwidth, *k*, of a directed chained graph is defined as

3.2 $$\begin{aligned} k=\min _{v_i\in {\overline{{{\mathcal {V}}}}}} k_i, \end{aligned}$$

where the minimum is over all initial vertices $v_i$ in the vertex set ${\overline{{{\mathcal {V}}}}}\subset {{\mathcal {V}}}$ that gives maximal chain length $\ell$. When *k* is the minimal lower bandwidth, the graph is said to be directed $\{\ell ,k\}$-chained.

The $\{\ell ,k\}$-chained structure is quite general. We conjecture that any weakly connected graph with *n* nodes is $\{\ell ,k\}$-chained for some $n\ge \ell >k\ge -1$. A small value of *k* indicates that information in the graph flows in a preferred direction, with small back propagation. This structure can be investigated by means of spanning trees as described in the section “Directed chained graphs and directed spanning trees”.

### Example 3.2

**Fig. 2:**
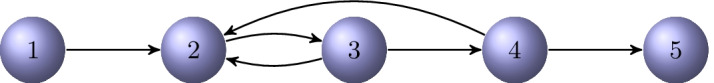
A directed $\{5,2\}$-chained graph ${{\mathcal {G}}}$ with initial vertex $v_1$

Consider the directed graph ${{\mathcal {G}}}$ shown in Fig. 2. It is a directed $\{5,2\}$-chained graph with initial vertex $v_1$. If one removes the edge from vertex $v_4$ to $v_2$, the graph becomes a directed $\{5,1\}$-chained graph with initial vertex $v_1$. If one continues by removing the edge from $v_3$ to $v_2$, then a directed 5-chained graph with the same initial vertex is obtained.

The adjacency matrix analogous to Eq. (3.1) for a directed $\{\ell ,k\}$-chained graph ${{\mathcal {G}}}= \{{{\mathcal {V}}},{{\mathcal {E}}}\}$ can be represented by a lower block Hessenberg matrix

3.3 $$\begin{aligned} A = \begin{bmatrix} A_{11} & A_{12} \\ A_{21}& A_{22} & A_{23} \\ \vdots & \vdots & \ddots & \ddots & \\ A_{k+1,1}& \vdots & & \ddots & \ddots \\ & A_{k+2,2} & & & \ddots & \ddots &\\ & & \ddots & & & A_{\ell -1,\ell -1} & A_{\ell -1,\ell } \\ & & & A_{\ell ,\ell -k} & \cdots & A_{\ell ,\ell -1} & A_{\ell ,\ell } \end{bmatrix}, \end{aligned}$$

when the nodes are suitably ordered. Here the block $A_{ij}$ represents edges that point from the vertex subset ${{\mathcal {V}}}_i$ to the vertex subset ${{\mathcal {V}}}_j$. All superdiagonal blocks $A_{i,i+1}$ are nonvanishing, because if all entries of the block $A_{i,i+1}$ were zero, then there would be no edges from the vertex subset ${{\mathcal {V}}}_i$ to vertices in the subset ${{\mathcal {V}}}_{i+1}$. But this would contradict the fact that the graph ${{\mathcal {G}}}$ is directed $\{\ell ,k\}$-chained.

If the minimal lower bandwidth, defined by Eq. (3.2), is $k=0$, then there is at least one edge from a node to another node in the same vertex subset. The adjacency matrix corresponding to such a graph is upper block bidiagonal when the nodes are suitably ordered. Similarly, a lower bandwidth $k=1$ indicates that when the nodes are suitably enumerated, the adjacency matrix can be represented by a block tridiagonal matrix. More generally, a small lower bandwidth (3.2) indicates that there only are edges between vertex subsets ${{\mathcal {V}}}_j$ with close indices.

The following result shows that for strongly connected directed $\{\ell ,k\}$-chained graphs, directed cycles will be observed if $k\ge 1$. For semi-connected or weakly connected directed graphs, cycles are not guaranteed to exist.

### Proposition 2

Let ${{\mathcal {G}}}=\{{{\mathcal {V}}},{{\mathcal {E}}}\}$ be a strongly connected directed $\{\ell ,k\}$-chained graph with vertex partition ${{\mathcal {V}}}={{\mathcal {V}}}_1 \cup \cdots \cup {{\mathcal {V}}}_{\ell }$. Assume there are no edges between vertices belonging to the same vertex set and that $k\ge 1$. Let $e_{j,i}\in {{\mathcal {E}}}$ represent a directed edge from vertex $v_j$ to $v_i$, where $v_i\in {{\mathcal {V}}}_i$ and $v_j\in {{\mathcal {V}}}_{i+s}$ for $1\le s\le k$. Then there exists at least one directed cycle that starts at $v_i$, contains the edge $e_{j,i}$, and ends at $v_i$. The possible minimum length of the directed cycle is $s+1$.

### Proof

Since the graph ${{\mathcal {G}}}$ is strongly connected and there are no edges between any nodes in the same vertex subset, the shortest possible directed path from vertex $v_i$ to $v_j$ has length *s* as shown below

$$\begin{aligned} v_i\rightarrow v_{i_1} \rightarrow \cdots \rightarrow v_{i_{s-1}} \rightarrow v_j, \end{aligned}$$

where $v_{i_t}\in {{\mathcal {V}}}_{i+t}$ for $t=1,2,\ldots ,s-1$. Combining this path with the edge $e_{j,i}$ determines a directed cycle of length $s+1$. $\square$

Identification of the $\{\ell ,k\}$-chained structure of a directed graph (if present) sheds considerable light on properties of the graph, including the presence of anti-communities. Anti-communities are vertex subsets ${{\mathcal {W}}}_i$, $i=1,2,\ldots ,q$, of ${{\mathcal {V}}}$ such that there are many fewer edges from nodes in ${{\mathcal {W}}}_i$ to nodes in ${{\mathcal {W}}}_i$, than from nodes in ${{\mathcal {W}}}_i$ to nodes in ${{\mathcal {W}}}_j$ for $j\ne i$. For instance, the node subsets ${{\mathcal {V}}}_j$ of an $\ell$-chained graph are anti-communities. Recent discussions on anti-community detection for undirected graphs can be found in Concas et al. (2020), Estrada and Knight (2015), Fasino and Tudisco (2017). There are several methods and measures that allow one to identify communities or clusters, such as the intra-cluster density which, for undirected graphs, is defined as the ratio of the number of internal edges and the number of all possible internal edges; see Fortunato (2010). An analogous density measure for computing the anti-community score for undirected graphs was introduced in Concas et al. (2021). Here, we extend this measure to directed $\{\ell ,k\}$-chained graphs.

### Definition 4

The anti-community score $\rho \in [0,1]$ for a node subset ${{\mathcal {V}}}_i$ of the node set ${{\mathcal {V}}}$ of a directed $\{\ell ,k\}$-chained graph is the ratio of the number of directed edges between the vertices in ${{\mathcal {V}}}_i$ and the total possible number of directed edges between them. An anti-community with score $\rho$ is said to be a $\rho$-anti-community.

We remark that the anti-community score aims at identifying an approximate anti-community as a node set for which $\rho$ takes a small value. A large value of $\rho$ does not necessarily identify a community, because it does not consider the connections between the nodes in ${{\mathcal {V}}}_i$ and those not contained in ${{\mathcal {V}}}_i$.

### Example 3.3

For directed $\ell$-chained graphs with node subset partitioning (2.2), the subsets ${{\mathcal {V}}}_i$, for $i=1,2,\ldots ,\ell$, are 0-anti-communities, because there are no internal edges. For a directed $\{\ell ,k\}$-chained graph described in Definition 2, the subset ${{\mathcal {V}}}_i$ has a positive anti-community score $\rho _i$ when it has internal edges. If $\rho _i$ is small, then the subset ${{\mathcal {V}}}_i$ may be considered as an approximate anti-community.

## Directed chained graphs and directed spanning trees

The chained structure of a spanning tree *T* for an undirected graph ${{\mathcal {G}}}$ is used in Concas et al. (2021) to determine a chained structure for a graph ${{\mathcal {G}}}$, if such a structure exists, and to approximate a graph without a chained structure by a graph with such a structure. In this section, we consider directed graphs that have directed spanning trees. We remark that not all directed graphs have a directed spanning tree. The directed spanning trees are employed to partition the node set ${{\mathcal {V}}}$ into subsets ${{\mathcal {V}}}_i$ that determine directed $\ell$-chained graphs; cf. (2.2). This approach to partition the node set ${{\mathcal {V}}}$ is applied to partitioning node sets of directed graphs that have a directed spanning tree, but do not possess a chained structure, and provides an approach to approximate a directed graph ${{\mathcal {G}}}$ without chained structure by a directed graph with chained structure.

We first briefly review results for undirected graphs. Let ${{\mathcal {G}}}=\{{{\mathcal {V}}},{{\mathcal {E}}}\}$ be an undirected graph. A spanning tree for ${{\mathcal {G}}}$ is a subgraph ${{\mathcal {T}}}=\{{{\mathcal {V}}},{{\mathcal {E}}}'\}$ that is a tree and contains all the vertices of ${{\mathcal {G}}}$; see, e.g., Concas et al. (2021), Deo (1974), Newman (2010). A spanning tree ${{\mathcal {T}}}$ is not uniquely determined by ${{\mathcal {G}}}$ and, in particular, depends on the chosen initial vertex of the tree, the so-called root.

When the graph ${{\mathcal {G}}}$ is directed, two different types of spanning directed trees, the out-tree (or arborescence) and the in-tree, can be defined; see Deo (1974). We will employ both these directed trees.

### Definition 5

An out-tree rooted at node $v_i$ for a directed graph ${{\mathcal {G}}}=\{{{\mathcal {V}}},{{\mathcal {E}}}\}$ is a subgraph ${{\mathcal {T}}}_\text {out}^i=\{{{\mathcal {V}}},{{\mathcal {E}}}'\}$ of ${{\mathcal {G}}}$ that is a tree with the same vertices as ${{\mathcal {G}}}$, and such that for every vertex $v_j$, for $j\ne i$, there is only one directed path starting at $v_i$ and ending at $v_j$ in the tree.

### Definition 6

An in-tree rooted at $v_i$ for a directed graph ${{\mathcal {G}}}=\{{{\mathcal {V}}},{{\mathcal {E}}}\}$ is a subgraph ${{\mathcal {T}}}_\text {in}^i=\{{{\mathcal {V}}},{{\mathcal {E}}}'\}$ of ${{\mathcal {G}}}$ that is a tree with the same vertices as ${{\mathcal {G}}}$, and such that for every vertex $v_j$, for $j\ne i$, there is only one directed path from $v_j$ to $v_i$ in the tree.

In an out-tree, information may flow from the root to each vertex in the graph, while in an in-tree information may flow from any vertex to the root. In the first case, the root is a good source of information for the nodes of the graph; in the second case, the root is a good receiver.

Out-trees and in-trees exist for every vertex of a directed graph only if the graph is strongly connected. Any vertex in a semi-connected graph belongs to an out-tree or an in-tree. This follows from Proposition 3 below. We remark that this property is not guaranteed to hold for a weakly connected graph.

### Proposition 3

Let ${{\mathcal {G}}}=\{{{\mathcal {V}}},{{\mathcal {E}}}\}$ be a semi-connected directed graph. Then the graph ${{\mathcal {G}}}$ has at least one out-tree and one in-tree.

### Proof

Let $v_i,v_j\in {{\mathcal {V}}}$ be arbitrary distinct vertices. Then either $v_i$ is connected to $v_j$, or $v_j$ is connected to $v_i$. Assume there is a directed path *P* from $v_i$ to $v_j$. If all the vertices of ${{\mathcal {G}}}$ except for $v_i$ and $v_j$ are on the path *P*, then *P* is an out-tree rooted at $v_i$ and an in-tree rooted at $v_j$.

Let *u* be a vertex of ${{\mathcal {G}}}$ that is not on the path *P*. Assume that there is neither a directed path from *u* to $v_i$ nor a directed path from $v_j$ to *u*; otherwise, we extend *P* by including *u* as a root. Then ${{\mathcal {E}}}$ contains directed paths from $v_i$ to *u* and from *u* to $v_j$. Therefore an out-tree rooted at $v_i$ and an in-tree rooted at $v_j$ are obtained. $\square$

### Proposition 4

Let ${{\mathcal {G}}}$ be a directed graph. If the vertex $v_i$ of ${{\mathcal {G}}}$ is the root of both an out-tree ${{\mathcal {T}}}_\text {out}^i$ and an in-tree ${{\mathcal {T}}}_\text {in}^i$ of ${{\mathcal {G}}}$, then the graph ${{\mathcal {G}}}$ is strongly connected.

### Proof

Let $v_i$ satisfy the assumption of the proposition. Then for any vertex $v_j$, $j\ne i$, there is a directed path from $v_i$ to $v_j$ and vice-versa. Hence, for every pair of vertices $(v_k, v_j)$, $k,j\ne i$, there is a directed path from $v_k$ to $v_j$ passing through $v_i$ and vice versa. It follows that the directed graph ${{\mathcal {G}}}$ is strongly connected. $\square$

Each directed spanning tree has a directed $\ell$-chained structure (2.2). For out-trees, the root of the tree is the only vertex in the first set ${{\mathcal {V}}}_1$ of the chained structure, and the partition of the vertex set ${{\mathcal {V}}}$ is determined by the relation between the vertices of the tree. Thus, the vertex set ${{\mathcal {V}}}_2$ contains the children of the root and, in general, the vertex set ${{\mathcal {V}}}_i$ contains the children of the vertices in ${{\mathcal {V}}}_{i-1}$, $i=2,3,\ldots ,\ell$. For an in-tree, the root belongs to the last vertex set ${{\mathcal {V}}}_\ell$, and the partition of the vertex set is determined by following the direction of the edges backwards until one reaches the set ${{\mathcal {V}}}_1$, which contains the leaves farthest away from the root.

### Remark 4.1

Let ${{\mathcal {G}}}=\{{{\mathcal {V}}},{{\mathcal {E}}}\}$, with ${{\mathcal {V}}}={{\mathcal {V}}}_1\cup {{\mathcal {V}}}_2\cup \cdots \cup {{\mathcal {V}}}_\ell$, be an $\ell$-chained graph. A directed out-tree for ${{\mathcal {G}}}$ does not exist, for example, if the first set ${{\mathcal {V}}}_1$ contains more than one node. Similarly, a directed in-tree does not exist when the last set ${{\mathcal {V}}}_\ell$ contains more than one node.

The process of generating directed spanning trees for a directed graph is illustrated in the following example.

### Example 4.1

**Fig. 3:**
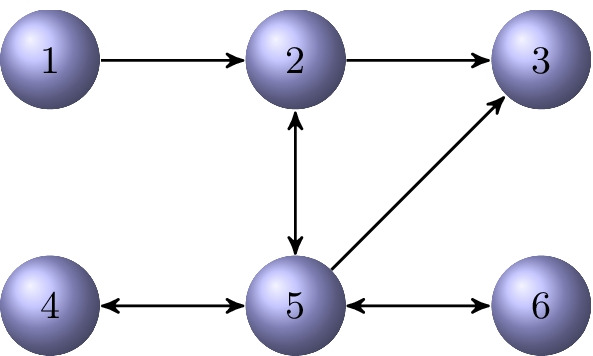
A directed graph $${{\mathcal {G}}}$$

**Fig. 4:**
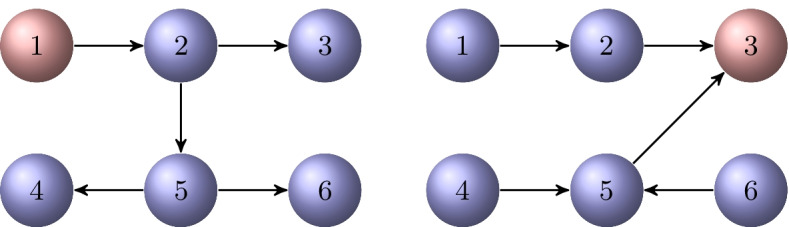
An out-tree ${{\mathcal {T}}}_\text {out}^1$ with root $v_1$ for the graph ${{\mathcal {G}}}$ in Fig. 3, and an in-tree ${{\mathcal {T}}}_\text {in}^3$ rooted at $v_3$

**Fig. 5:**
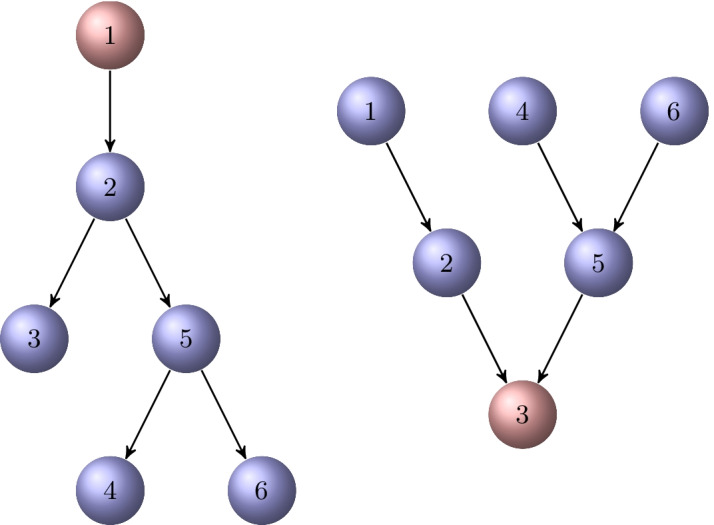
The directed chained structure of the spanning trees ${{\mathcal {T}}}_\text {out}^1$ and ${{\mathcal {T}}}_\text {in}^3$ in Fig. 4

Consider the directed graph ${{\mathcal {G}}}$ shown in Fig. 3. It is semi-connected. The out-tree ${{\mathcal {T}}}_\text {out}^1$ and the in-tree ${{\mathcal {T}}}_\text {in}^3$ rooted at $v_1$ and $v_3$, respectively, are displayed in Fig. 4. These are the only out-trees and in-trees for the graph ${{\mathcal {G}}}$. Their directed chained structure is illustrated in Fig. 5.

The chained structure of a directed spanning tree ${{\mathcal {T}}}$ of ${{\mathcal {G}}}$ can be used to detect, or approximate, the directed chained structure of ${{\mathcal {G}}}$. The chained structure of ${{\mathcal {G}}}$ might not be unique, as it depends on the starting vertex and the directed spanning tree ${{\mathcal {T}}}$.

### Definition 7

Let ${{\mathcal {T}}}=\{{{\mathcal {V}}},{{\mathcal {E}}}'\}$ be an out-tree (or in-tree) for the graph ${{\mathcal {G}}}$. A directed $\ell$-chained vertex set decomposition for ${{\mathcal {T}}}$ is said to be a directed $\ell$-chained vertex set decomposition for ${{\mathcal {G}}}$. We will refer to leaves of ${{\mathcal {T}}}$ as leaves of ${{\mathcal {G}}}$.

### Example 4.2

Consider the partition of the directed tree ${{\mathcal {T}}}_\text {out}^1$ in Example 4.1. Starting with the vertex $v_1$, we obtain the vertex sets ${{\mathcal {V}}}_1=\lbrace v_1\rbrace$, ${{\mathcal {V}}}_2=\lbrace v_2\rbrace$, ${{\mathcal {V}}}_3=\lbrace v_3, v_5 \rbrace$, and ${{\mathcal {V}}}_4=\lbrace v_4,v_6\rbrace$. Thus, $\ell =4$; see Fig. 5. The graph may have other directed $\ell$-chained partitions that are not determined by using spanning trees. For example, starting with vertices $v_1, v_4$, we obtain the partition ${{\mathcal {V}}}_1=\lbrace v_1,v_4\rbrace$, ${{\mathcal {V}}}_2=\lbrace v_2,v_5\rbrace$, and ${{\mathcal {V}}}_3=\lbrace v_3, v_6 \rbrace$, and $\ell =3$.

Now consider the in-tree ${{\mathcal {T}}}_\text {in}^3$ in Fig. 4. In this case, ${{\mathcal {V}}}_1=\lbrace v_1, v_4, v_6\rbrace$, ${{\mathcal {V}}}_2=\lbrace v_2, v_5 \rbrace$, ${{\mathcal {V}}}_3=\lbrace v_3\rbrace$, and $\ell =3$.

We have already mentioned that some semi-connected graphs may not allow an $\ell$-chained partitioning for an arbitrarily chosen initial vertex. For example, vertex $v_2$ in Example 4.1 neither can be the root of an out-tree nor of an in-tree that spans the graph.

Let ${{\mathcal {D}}}={{\mathcal {E}}}\setminus {{\mathcal {E}}}'$ be the set of the edges in ${{\mathcal {G}}}$ that are not in ${{\mathcal {T}}}$, and let $C({{\mathcal {T}}})$ denote the graph obtained by adding the edges in ${{\mathcal {D}}}$ to the spanning tree ${{\mathcal {T}}}$. The graph $C({{\mathcal {T}}})$ coincides with ${{\mathcal {G}}}$ and inherits the chained structure of ${{\mathcal {T}}}$.

### Definition 8

A directed graph ${{\mathcal {G}}}$ is said to be compatible with a spanning tree ${{\mathcal {T}}}$ if all the edges in ${{\mathcal {D}}}$ are compatible with the chained structure of ${{\mathcal {T}}}$, that is, if for each edge $e_j\in {{\mathcal {D}}}$ there is an index $2\le i\le \ell -1$ such that $e_j$ connects a vertex in ${{\mathcal {V}}}_i$ to a vertex in ${{\mathcal {V}}}_{i+1}$.

If ${{\mathcal {G}}}$ is compatible with ${{\mathcal {T}}}$, then the graph ${{\mathcal {G}}}=C({{\mathcal {T}}})$ is directed $\ell$-chained. If instead there is at least one edge connecting a vertex in ${{\mathcal {V}}}_i$ to a vertex in ${{\mathcal {V}}}_{i-k}$, for $i=k+1,k+2,\ldots ,\ell$, and $k\ge 0$ is the maximal number with this property, then the graph ${{\mathcal {G}}}=C({{\mathcal {T}}})$ is directed $\{\ell ,k\}$-chained.

**Fig. 6:**
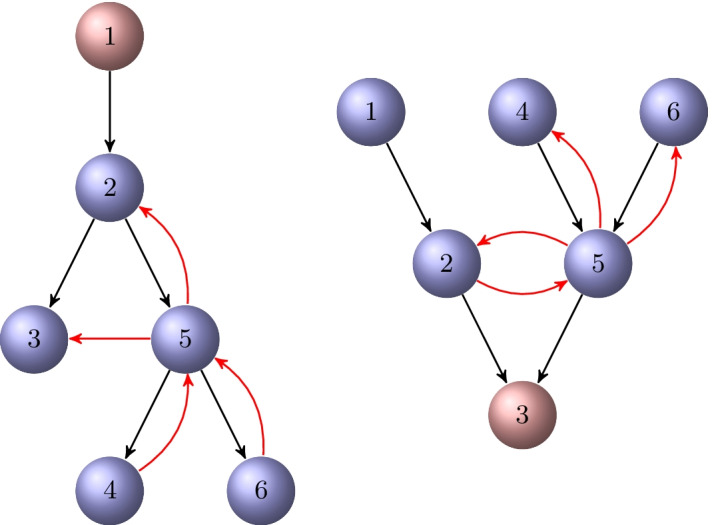
The directed graphs $C({{\mathcal {T}}}_\text {out}^1)$ and $C({{\mathcal {T}}}_\text {in}^3)$ corresponding to the directed spanning trees in Fig. 5. The edges in ${{\mathcal {D}}}={{\mathcal {E}}}\setminus {{\mathcal {E}}}'$ added to the trees are drawn in red

The graphs $C({{\mathcal {T}}}_\text {out}^1)$ and $C({{\mathcal {T}}}_\text {in}^3)$, obtained by adding the missing edges to the spanning trees of Fig. 5, are displayed in Fig. 6. The former graph is $\{4,1\}$-chained and the latter one is $\{3,1\}$-chained.

## Position centrality and some applications

The notion of position centrality for vertices of an undirected network was introduced in Concas et al. (2021). It is a generalization of closeness centrality. This section generalizes position centrality to directed graphs by defining the in-position and out-position centralities of a node. The in-closeness centrality of a node measures how close this node is to those it is receiving information from, while the out-closeness centrality of a node shows how close the node is to the nodes it is sending information to.

Let $(\#{{\mathcal {V}}}_i)$ denote the number of vertices in the set ${{\mathcal {V}}}_i$.

### Definition 9

Let us assume that an out-tree ${{\mathcal {T}}}_\text {out}=\{{{\mathcal {V}}},{{\mathcal {E}}}'\}$ rooted at the node *v* for the directed graph ${{\mathcal {G}}}$ exist. Moreover, let ${{\mathcal {V}}}_1,{{\mathcal {V}}}_2,\ldots ,{{\mathcal {V}}}_\ell$ be the directed $\ell$-chained structure, starting at vertex *v*, determined by the tree. For a fixed $p\in {{\mathbb {R}}}$, the *out-position centrality* of *v* is defined as

$$\begin{aligned} P^\text {out}_p(v) = \sum _{k=1}^{\ell -1} k (\#{{\mathcal {V}}}_{k+1})^p. \end{aligned}$$

We refer to a vertex $v_c$ with the smallest out-position centrality as a *p*-out-center vertex.

### Definition 10

Let us assume that an in-tree ${{\mathcal {T}}}_\text {in}=\{{{\mathcal {V}}},{{\mathcal {E}}}'\}$ rooted at the node *v* for the directed graph ${{\mathcal {G}}}$ exist. Moreover, let ${{\mathcal {V}}}_1,{{\mathcal {V}}}_2,\ldots ,{{\mathcal {V}}}_\ell$ be the directed $\ell$-chained structure, ending at vertex *v*, determined by the tree. For a fixed $p\in {{\mathbb {R}}}$, the *in-position centrality* of *v* is defined as

$$\begin{aligned} P^\text {in}_p(v) = \sum _{k=1}^{\ell -1} k (\#{{\mathcal {V}}}_{\ell -k})^p. \end{aligned}$$

We refer to a vertex $v_c$ with the smallest in-position centrality as a *p*-in-center vertex.

The in/out-position centralities depend on the spanning tree chosen. They can be defined for every node only if the directed graph is strongly connected. The out-center vertex can be described as an “information transfer station”, such that it can “easily” send information to all the other vertices in the graph. A similar interpretation holds for the in-center vertex, which acts as an information sink. The following example illustrates how the in/out-position centralities of a vertex can be computed by using the chained structures starting from the vertex.

### Example 5.1

**Fig. 7:**
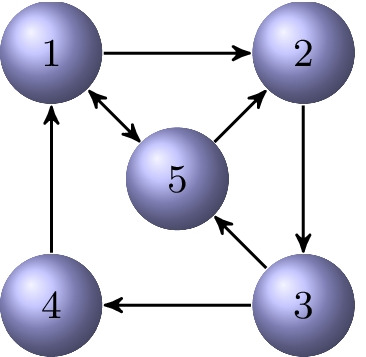
A strongly connected directed graph ${{\mathcal {G}}}$

Consider the strongly connected directed graph ${{\mathcal {G}}}$ in Fig. 7. To compute the out-position centrality of vertex $v_3$, we identify an out-tree rooted at $v_3$ letting ${{\mathcal {V}}}_1=\{v_3\}$, ${{\mathcal {V}}}_2=\{v_4,v_5\}$, and ${{\mathcal {V}}}_3=\{v_1,v_2\}$. The 1-out-position centrality of vertex $v_3$ is

$$\begin{aligned} P_1^{out}(v_3)=1\cdot 2+2\cdot 2=6, \end{aligned}$$

while $$P_{1/2}^{out}(v_3)=4.24$$ and $$P_{5}^{out}(v_3)=96$$.

We turn to the in-position centrality of vertex $v_2$. Consider the in-tree rooted at $v_2$ with vertex set partitioning ${{\mathcal {V}}}_1=\{v_3,v_4\}$, ${{\mathcal {V}}}_2=\{v_1,v_5\}$, and ${{\mathcal {V}}}_3=\{v_2\}$. We have

$$\begin{aligned} P_1^{in}(v_2)=1\cdot 2+2\cdot 2=6, \quad P_{1/2}^{in}(v_2)=4.24, \quad P_{5}^{in}(v_3)=96. \end{aligned}$$

Since the graph ${{\mathcal {G}}}$ is strongly connected, we can compute the in/out-position centralities for all the other vertices similarly. When $p=\frac{1}{2}$ and $p=1$, the vertex $v_3$ has the smallest out-position centrality. This indicates that $v_3$ is the out-center vertex. The in-center vertices are $v_2$ and $v_5$ for $p=\frac{1}{2}$ and $p=1$. When $p=5$, the out-center vertices are $v_1$ and $v_5$, while the in-center vertex is $v_1$.

In a semi-connected directed graph, the vertices can be divided into three subsets: ${{\mathcal {O}}}$, which contains vertices connected to every other vertex in the network, ${{\mathcal {I}}}$, whose elements are vertices to which every vertex can send information, and ${{\mathcal {M}}}$, which contains intermediate vertices. There may be a non-empty intersection between the sets ${{\mathcal {O}}}$ and ${{\mathcal {I}}}$. Out-position centrality is defined only for vertices in ${{\mathcal {O}}}$, while in-position centrality can be computed for vertices in ${{\mathcal {I}}}$. Since every vertex belongs to at least one spanning tree, semi-connected graphs are directed $\ell$-chained or directed $\{\ell ,k\}$-chained.

Vertices for weakly connected directed graphs also can be divided into the above three subsets ${{\mathcal {O}}}$, ${{\mathcal {I}}}$, and ${{\mathcal {M}}}$. However, the sets ${{\mathcal {O}}}$ and ${{\mathcal {I}}}$ may both be empty, since out/in-trees are not guaranteed to exist. Hence, weakly connected directed graph may not possess a chained structure.

## Some examples

This section describes a few examples concerned with directed graphs. For each graph, we analyze the presence of anti-communities by identifying its directed chained structure. The out/in-center vertices are identified by computing the smallest out/in-position centralities. Knowledge of the chained structure is beneficial in the following contexts:

- Information dissemination in a social network: we are interested in determining directed $\ell$-chained or directed $\{\ell ,k\}$-chained structures with initial vertex (center vertex) such that information from this node can reach all other individuals in the least amount of time, where we assume that the time is proportional to the path length. Similarly, we may be interested in determining which individual(s) can collect information from all other vertices in the least amount of time. Moreover, in a directed $\{\ell ,k\}$-chained graph, the presence of an edge $e_{j,i}$ from vertex $v_j\in {{\mathcal {V}}}_j$ to vertex $v_i\in {{\mathcal {V}}}_i$, for $i<j$, indicates the possibility of feedback of the information from $v_j$ to $v_i$. The minimal lower bandwidth *k* shows the minimal length of the path from $v_j$ to $v_i$.
- Prevention of the spread of an infectious disease: let the edges of a directed chained graph represent the spread of an infectious disease among subjects that are represented by nodes. An edge $e_{j,i}$ from vertex $v_j\in {{\mathcal {V}}}_j$ to vertex $v_i\in {{\mathcal {V}}}_i$, for $i<j$, represents a secondary infection of $v_i$ from $v_j$. It is reasonable to prevent the spread of disease by detecting and possibly eliminating the out-center vertex. In the context of COVID-19, it is important that out-center vertices be vaccinated. Similarly, it can be important to protect an in-center vertex from infection from other nodes. Vaccination may be one way to achieve this.

To graphically illustrate the chained structures revealed by the model discussed above, we first consider the following two small directed graphs:

- ibm32 (32 vertices, 126 edges): collected from the IBM 1971 conference advertisement. After removing self-loops, the graph has 94 edges. It is available at https://sparse.tamu.edu/HB/ibm32/.
- n2c6b10, short for JGD_Homology/n2c6-b10 (306 vertices, 330 edges): simplicial complexes from homology by Volkmar Welker. There are 329 edges after removing the self-loop. The graph is available at https://www.cise.ufl.edu/research/sparse/matrices/list_by_dimension.html.

These networks are not social networks, but nevertheless will be seen to have structure that can be studied with the concepts introduced in the present paper. The network n2c6b10 is represented by a weighted graph. We consider the corresponding unweighted graph obtained by setting all weights to 1.

**Fig. 8:**
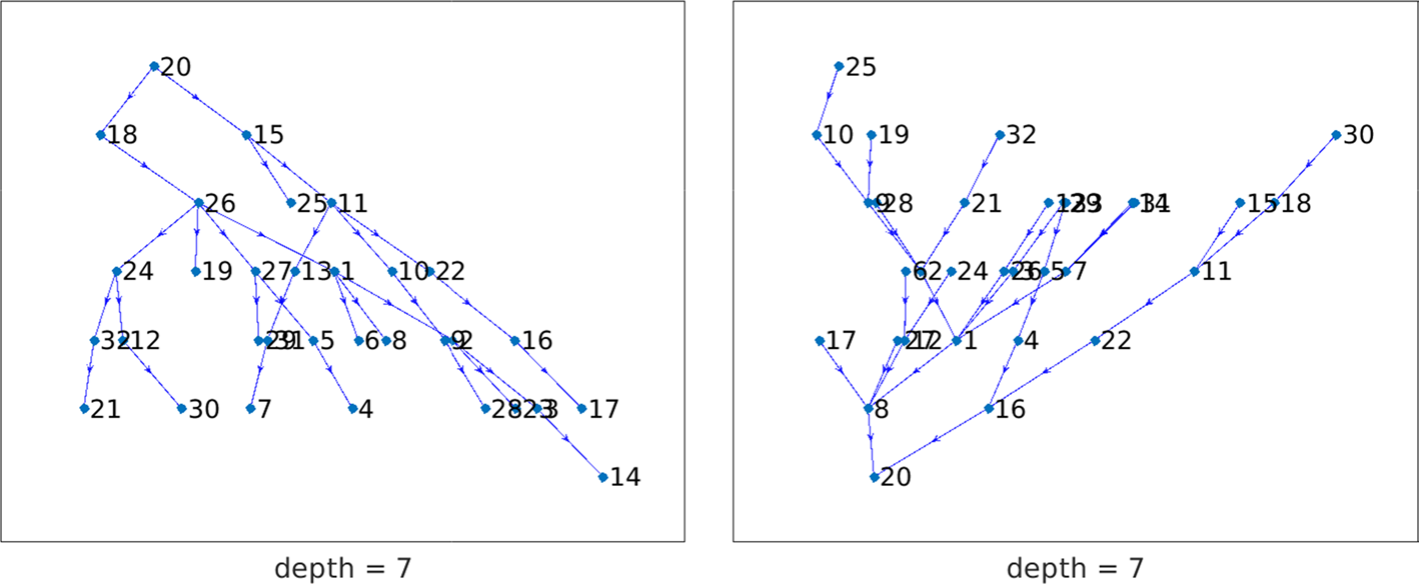
Network ibm32: the out-tree (left) and the in-tree (right) rooted at vertex $v_{20}$. These trees have maximal chain length $\ell =7$

**Fig. 9:**
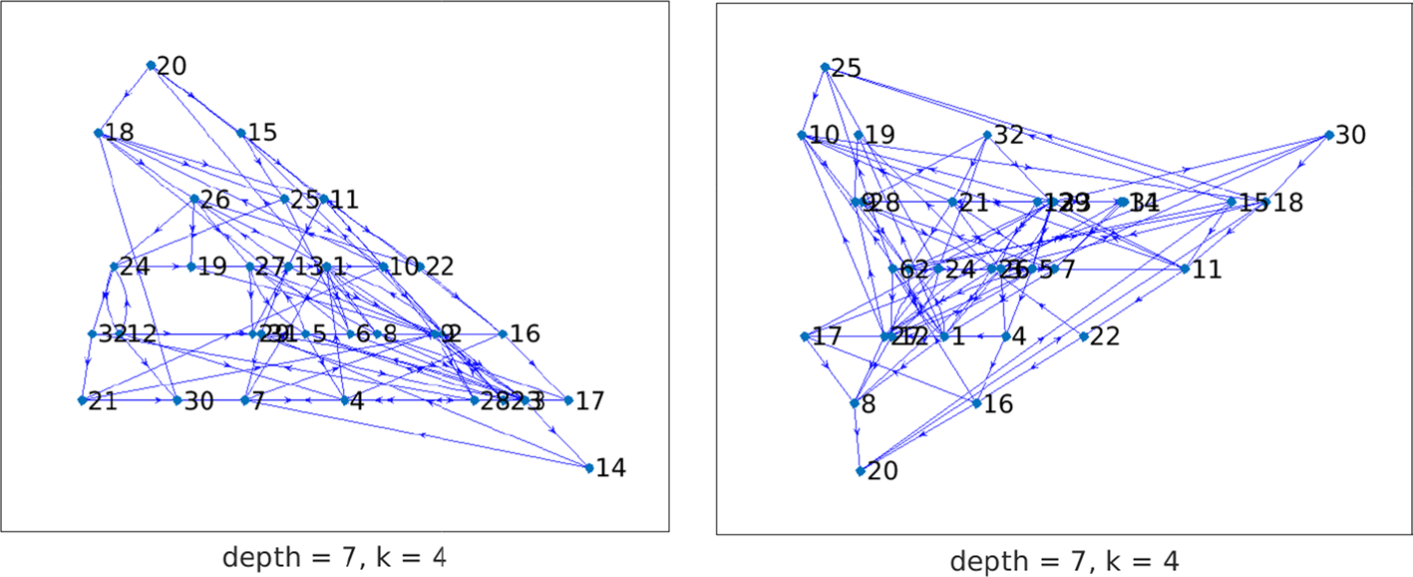
Network ibm32: Both the sets $C({{\mathcal {T}}}_\text {out}^{20})$ (left) and $C({{\mathcal {T}}}_\text {in}^{20})$ (right) has $\{\ell ,k\}$-chained structure with $k=4$

Figure 8 displays the out-tree ${{\mathcal {T}}}_\text {out}^{20}$ (left) and the in-tree ${{\mathcal {T}}}_\text {in}^{20}$ (right) for the ibm32 network. Both trees are rooted at vertex $v_{20}$ of the graph and have maximal chained structure length $\ell =7$. The set $C({{\mathcal {T}}}_\text {out}^{20})$, which contains the additional edges that are not in ${{\mathcal {T}}}_\text {out}^{20}$, is shown on the left-hand side of Fig. 9. It has $\{\ell ,k\}$-chained structure with minimal lower bandwidth $k=4$, that is, edges in $C({{\mathcal {T}}}_\text {out}^{20})$ from vertices in the subset ${{\mathcal {V}}}_i$ are allowed to point to vertices in the subsets ${{\mathcal {V}}}_{i-4},\ldots ,{{\mathcal {V}}}_i,{{\mathcal {V}}}_{i+1}$ for $i=5,\ldots ,\ell$. The right-hand side of Fig. 9 displays the graph $C({{\mathcal {T}}}_\text {in}^{20})$ with minimal lower bandwidth $k=4$. We conclude that the graph ibm32 is directed $\{7,4\}$-chained with initial vertex $v_{20}$. Consider the chained structure determined by the in-tree ${{\mathcal {T}}}_\text {in}^{20}$. There are four 0-anti-communities (the first subset ${{\mathcal {V}}}_1$ contains only one vertex) and 3 anti-communities with scores $\rho _3=0.10$, $\rho _4=0.11$, and $\rho _5=0.06$. Moreover, since both the out-tree and in-tree are rooted at vertex $v_{20}$, it follows from Theorem 4 that the graph is strongly connected.

**Fig. 10:**
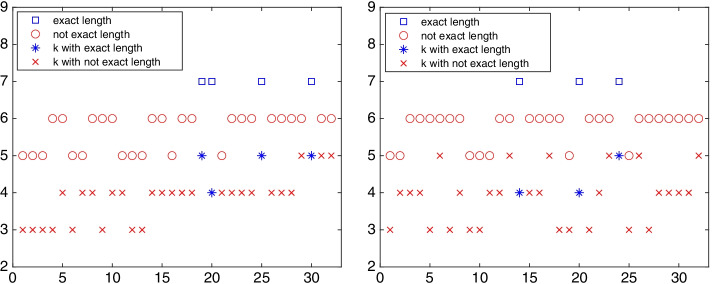
Network ibm32: the chain length and lower bandwidth of the $\{\ell ,k\}$-chained structure determined by the out-tree (left) and the in-tree (right) rooted at each vertex $v_j$, $j=1,2,\ldots ,32$

Starting from each vertex, an out-tree and an in-tree are constructed and their associated chained structures are determined. Figure 10 displays the chain length $\ell$ and the lower bandwidth *k* of the $\{\ell ,k\}$-chained structure associated with the out-tree (left) and the in-tree (right) rooted at each vertex of the graph ibm32. The property of “exact length” in the legend represents the maximal chain length and the property “not exact length” indicates that the chain length is not maximal. The symbol $\circ$ in the figure displays the chain length of each structure as a function of the initial vertex $v_j$, $j=1,2,\ldots ,32$. When the chain length is maximal, we use the symbol $\square$.

The symbols $*$ and $\times$ display the lower bandwidth $k_j$ of the chained structure with initial node $v_j$ for $j=1,2,\ldots ,32$; see Definition 2. The lower bandwidths of the chained structure with the maximal chain length are displayed by $*$ symbols; for the other chained structures, the symbol is $\times$. Among the lower bandwidths associated with the maximal chain length, the smallest $k_j$ is the minimal lower bandwidth. Hence, Fig. 10 shows the maximal chain length to be $\ell =7$ and the minimal lower bandwidth $k=4$. It can be seen that the maximal chain length and the minimal lower bandwidth are not achieved for each starting or ending vertex.

**Fig. 11:**
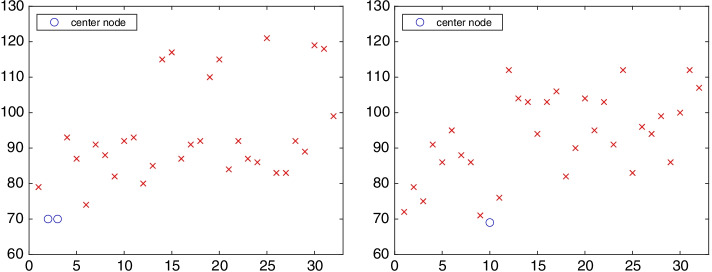
Network ibm32: out-position centrality for $p=1$ (left) and in-position centrality for $p=1$ (right) for each vertex $v_k$, $k=1,2,\ldots ,32$. The out-center vertices are $v_2$, $v_3$, and the in-center vertex is $v_{10}$

The left-hand side of Fig. 11 displays the 1-out-position centrality, i.e., the out-position centrality for $p=1$, of each vertex of graph ibm32. The 1-in-position centrality for each vertex is shown on the right-hand side of Fig. 11. The out-center vertices of the graph are $v_2$ and $v_3$, and the in-center vertex is $v_{10}$.

**Fig. 12:**
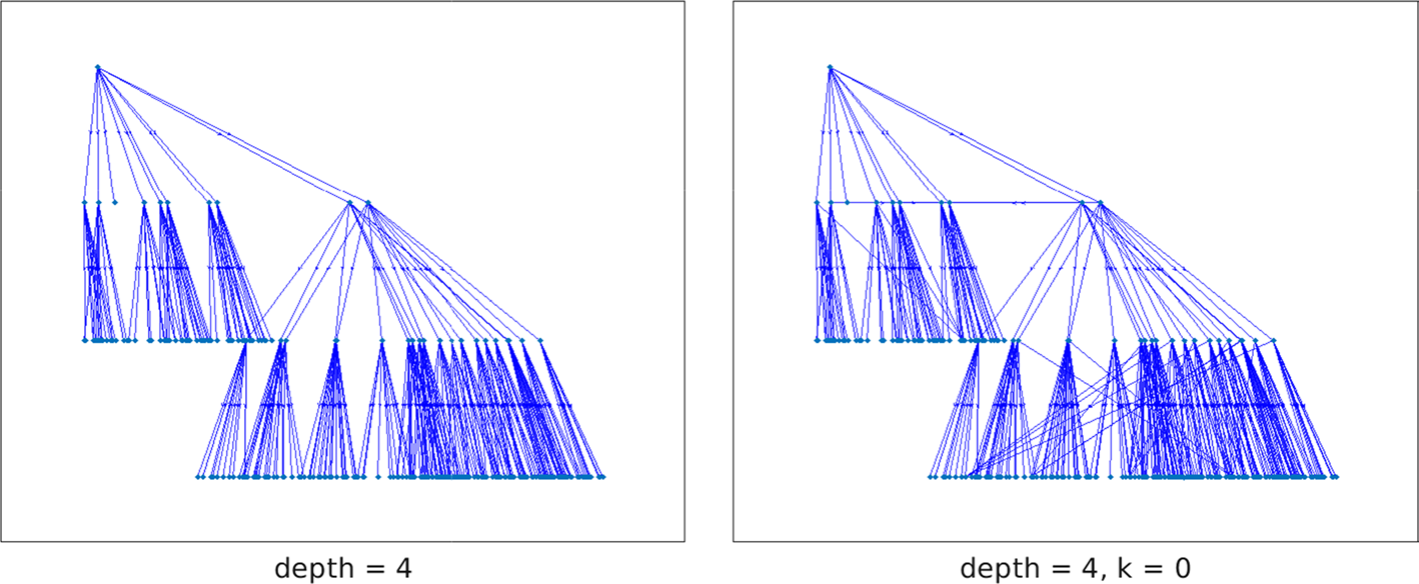
Network n2c6b10: out-tree ${{\mathcal {T}}}_\text {out}^{28}$ and graph $C({{\mathcal {T}}}_\text {out}^{28})$

Figure 12 displays the out-tree ${{\mathcal {T}}}_\text {out}^{28}$ with maximal chain length and its corresponding graph $C({{\mathcal {T}}}_\text {out}^{28})$ for the graph n2c6b10. We note that this graph is directed $\{4,0\}$-chained with initial vertex $v_{28}$. Since the out-tree ${{\mathcal {T}}}_\text {out}^{28}$ is the only spanning tree of n2c6b10, the initial vertex is the out-center vertex and the graph is semi-connected. The graph n2c6b10 has three 0-anti-communities and the node subset ${{\mathcal {V}}}_2$ is an anti-community with $\rho _2=0.04$.

**Fig. 13:**
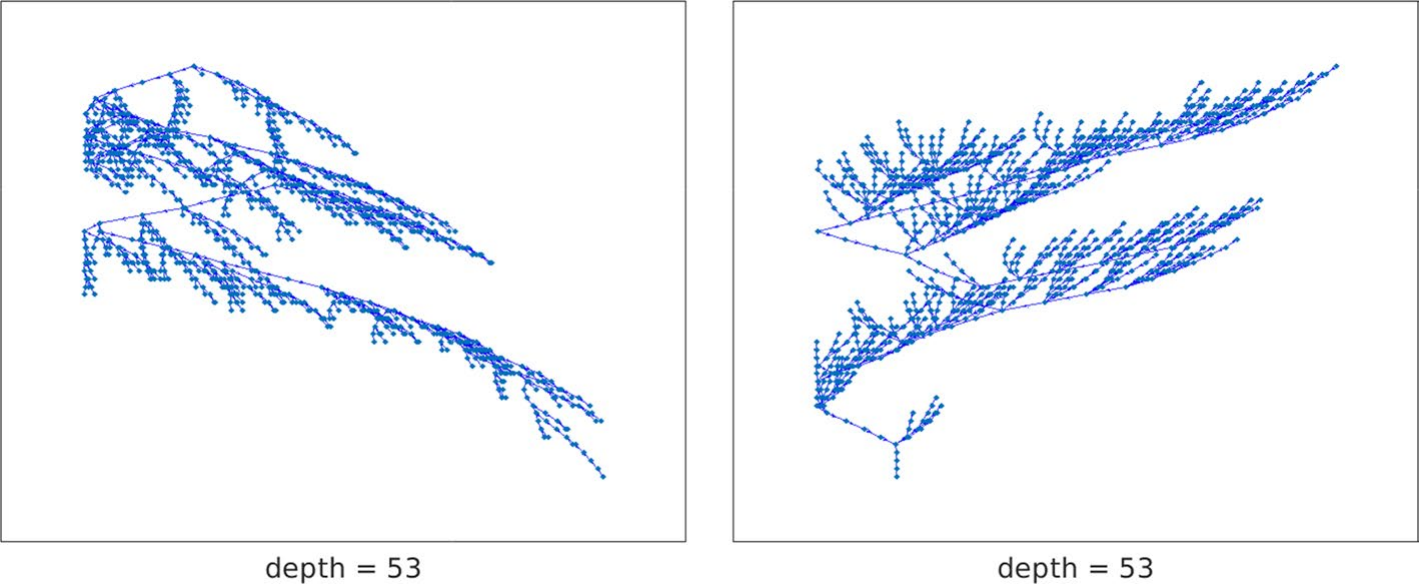
Network gre (1107 vertices): out-tree with root vertex 808 (left), and in-tree ending at vertex 644 (right) with maximal chained structure length

**Fig. 14:**
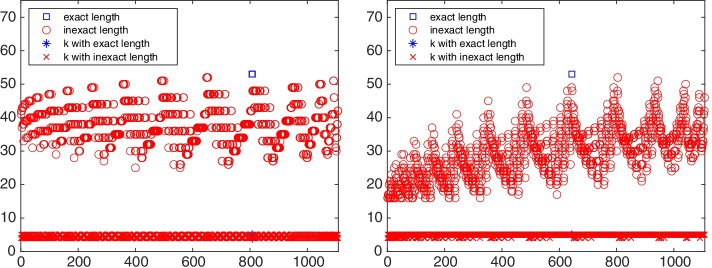
Network gre (1107 vertices): the chain length and lower bandwidth of the chain-like structure determined by out-trees (left) and in-trees (right) rooted at each vertex

We now determine the directed chained-like structure and center vertices for the medium-sized directed graph gre_1107 with 1107 vertices and 5664 edges. This graph arises from simulation studies in computer systems and is available at https://math.nist.gov/MatrixMarket/data/Harwell-Boeing/grenoble/gre_1107.html. We refer to the graph as gre. After removing self-loops, this graph has 4557 edges. The out-tree ${{\mathcal {T}}}_\text {out}^{808}$ and in-tree ${{\mathcal {T}}}_\text {in}^{644}$ with maximal chained structure length are displayed on the left and the right of Fig. 13, respectively. The graph $C({{\mathcal {T}}}_\text {out}^{808})$ is $\{53,4\}$-chained and the graph $C({{\mathcal {T}}}_\text {in}^{644})$ has a $\{53,5\}$-chained structure. The chain length and lower bandwidth of $\{\ell ,k\}$-chained structures starting and ending at each vertex of the graph gre are shown in Fig. 14. Only one directed out-tree, ${{\mathcal {T}}}_\text {out}^{808}$, and one directed in-tree, ${{\mathcal {T}}}_\text {in}^{644}$, are found to have maximal chain length. Their corresponding $\{\ell ,k\}$-chained structures have minimal lower bandwidth $k=4$ and $k=5$, respectively; this is not visible in the figure because of the density of the symbols. Hence, the graph gre is a directed $\{53,4\}$-chained graph with initial vertex $v_{808}$. It has five 0-anti-communities and 48-anti-communities with the minimal score $\rho =0.01$ and maximal score $\rho =0.5$. The graph gre is strongly connected since each vertex has both out-trees and in-trees.

**Fig. 15:**
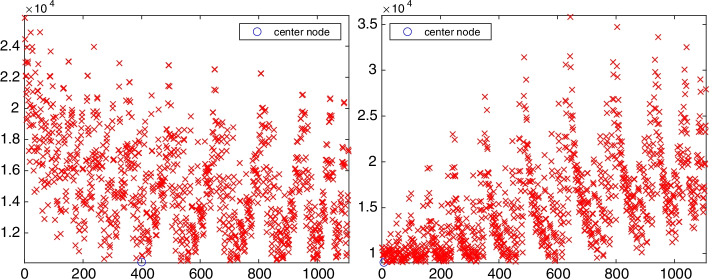
Network gre (1107 vertices): 1-out-position centrality (left) and 1-in-position centrality (right) for each vertex. The out-center vertex is $v_{400}$ and the in-center vertex is $v_{7}$

The 1-out-position centrality and 1-in-position centrality of each vertex of the graph gre are shown in Fig. 15. The out-center vertex is $v_{400}$ with 1-out-position centrality $P^\text {out}_1(v_{400})=10129$, and the in-center vertex is $v_7$ with 1-in-position centrality $P^\text {in}_1(v_7)=9030$.

For the previous test networks, of small to medium dimension, it was possible to determine both a spanning out-tree and an in-tree. Now, we investigate the presence of such spanning trees in larger networks, some of which are extracted from the Stanford Large Network Dataset Collection (SNAP) (Leskovec and Krevl 2014). The networks are the following

- twitter (3656 vertices, 188,712 edges) available from http://wiki.gephi.org/index.php/Datasets, reproduces the connections of some part of the Twitter social network;
- wikivote (8297 vertices, 103,690 edges). The nodes in the network represent Wikipedia users and a directed edge from node *i* to node *j* represents that user *i* voted on user *j* in an administrator election (Leskovec and Krevl 2014);
- gnutella (10,879 vertices, 39,994 edges) is the p2pGnutella04 network from Leskovec and Krevl (2014);
- foldoc (13,380 vertices, 120,700 edges) is an on-line searchable dictionary http://foldoc.org/: an edge from term *i* to term *j* exists in the network if in the FOLDOC dictionary the term *j* is used to describe the meaning of term *i*. The network is available at http://vlado.fmf.uni-lj.si/pub/networks/data/.
- math (13,840 vertices, 195,330 edges) is available from Leskovec and Krevl (2014) and represents the interactions on the stack exchange web site Math Overflow https://mathoverow.net/. In particular, a direct edge is present between node *i* and node *j* if user *i* commented on user *j*’s answer.

Table 1 displays, for each network, if a spanning out/in-tree exists, and the values of $\ell$ and *k* in the corresponding $\{\ell ,k\}$-chained structure.

Three of the above networks admit either a spanning out-tree or an in-tree. Two of them do not, so we eliminated the out/in dangling nodes, that is, vertices that do not have incoming edges or outgoing edges, respectively. This pre-processing is reflected in a different number of nodes in columns 3 and 5, than in column 2. The absence of dangling nodes is a necessary condition for the existence of an out/in spanning tree, but not sufficient, as the results in Table 1 confirm. The results show that there are real-world networks resulting from important applicative settings that have a directed spanning tree and that inherit from it a chained structure.

**Table 1:** The structure of a few large networks

Network	Nodes	Out-tree		In-tree	
		Nodes	$\{\ell ,k\}$	Nodes	$\{\ell ,k\}$
twitter	3656	3656	$\{13,8\}$	3656	No tree
wikivote	8297	1368	$\{10,9\}$	5162	No tree
gnutella	10879	4889	$\{26,19\}$	4353	$\{12,7\}$
foldoc	13380	13291	No tree	13291	$\{16,13\}$
math	13840	3460	No tree	3460	$\{9,5\}$

We now analyze in more detail the network twitter. To illustrate the different center nodes determined by varying the value of *p* in Definitions 9 and 10, we analyzed this graph for the *p* values reported in the first column of Table 2. It turns out that no vertex admits an in-tree, while most of the nodes (3485) have an out-tree. The table shows that different out-center vertices are identified when *p* varies, even if there is some stability for *p* between 0 and 1. Both the depth $\ell$ and the minimal lower bandwidth *k* of the corresponding $\{\ell ,k\}$-chained structure can be seen to grow with *p*.

**Table 2:** Network twitter (3656 vertices): in the upper part of the table, we report the *p*-out-center and the *p*-in-center vertices for different values of *p*; in the lower part, we show the center vertices identified by other centrality measures

*p*	Out-center	$\{\ell ,k\}$
$-1$	1768	$\{9,7\}$
0.1, 0.5, 1	1324	$\{9,7\}$
5	1990	$\{10,7\}$
10	1006	$\{11,9\}$
Degree	751	$\{10,8\}$
Betweenness	1756	$\{10,9\}$
PageRank	2356	$\{12,10\}$
HITS (hubs)	751	$\{10,8\}$
Hub centrality	751	$\{10,8\}$

For each vertex we also report the depth $\ell$ of the associated spanning tree and the minimal lower bandwidth *k* of the corresponding $\{\ell ,k\}$-chained structure

In the same table, we also report the out-center nodes identified by other well-known centrality measures. The out-degree, betweenness centrality (Newman 2010), PageRank (Page et al. 1999), and hubs score from HITS (Kleinberg 1999), have been computed by the centrality function of Matlab. The hub-centrality (Benzi et al. 2013) has been computed by the hubauth package, developed in Baglama et al. (2014) and available at https://bugs.unica.it/cana/software/.

Table 2 confirms the well-known fact that centrality measures often disagree, making it hard to judge which result is the best. The table also points out that trees rooted at the vertices with largest position centralities tend to identify chained structures with a smaller depth $\ell$ and bandwidth *k* than trees rooted at nodes considered important with respect to other measures. Table 2 illustrates that when using position centrality, we are able to identify chained structure with the smallest $\ell$ and *k* values.

**Fig. 16:**
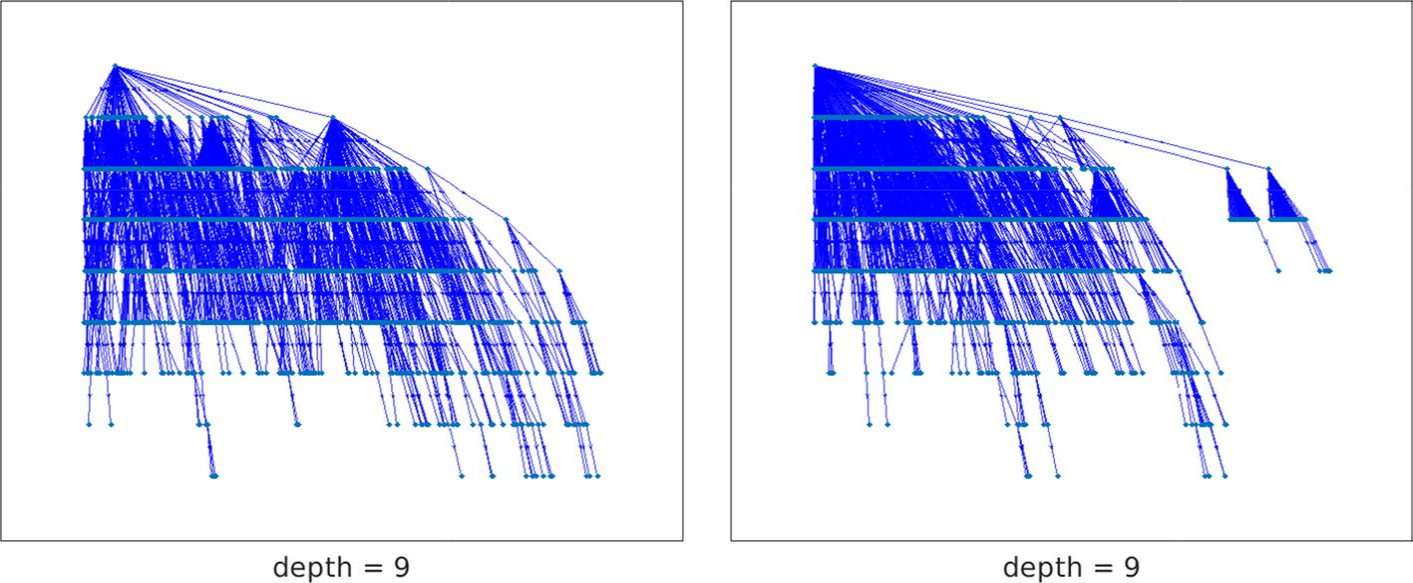
Network twitter (3656 vertices): out-trees with root vertices 1768 (left) and 1324 (right)

Figure 16 displays the spanning trees rooted at the first two out-center nodes of Table 2. It is evident that the tree corresponding to the larger value of *p*, shown on the right, produces a chained structure for which the cardinality of the ${{\mathcal {V}}}_j$ sets with small *j* is larger at the beginning of the sequence than for the tree on the left; the cardinality of the sets in the tree on the left are more balanced. However, it is difficult to understand the effect of this parameter on the choice of the center nodes without knowledge of the identity and history of the people defining the vertices. The transportation network analyzed in the next section aims to clarify the meaning of *p*-out-center nodes, as well as to compare position centrality to other centrality measures.

**Fig. 17:**
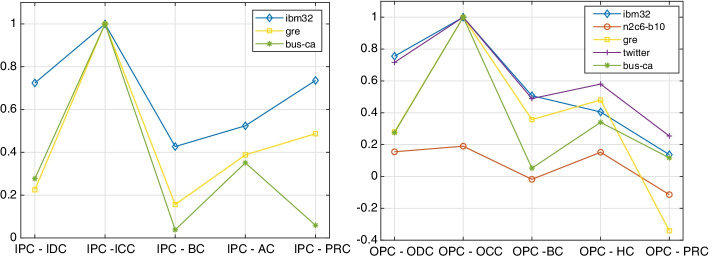
Kendall rank correlation coefficient between the in/out position centrality (IPC/OPC) and degree (IDC/ODC), closeness (ICC/OCC), betwenness (BC), hub/authority (HC/AC), and PageRank (PRC) centralities. The graph on the left concerns the incoming connections, the one on the right the outgoing ones. The computation has been performed for all the networks considered in the paper

To gain some insight into how the centrality measures considered in this section are related, we computed the Kendall rank correlation coefficient between the in/out position centrality with $p=1$ (IPC/OPC) and degree (IDC/ODC), closeness (ICC/OCC), betwenness (BC), hub/authority (HC/AC), and PageRank (PRC) centralities. The comparison has been performed on the ibm32, n2c6b10, gre, twitter, and bus-ca networks. The last network will be discussed in the next section.

The results are displayed in Fig. 17. The networks n2c6b10 and twitter do not appear in the graph on the left, because they have no in-center nodes; there is only one out-center node in n2c6b10, so for this data set the comparison is meaningless, as it is clear in the graph on the right. As it is expected, the Kendall coefficients of position centrality and closeness centrality for both incoming and outcoming connections are 1, meaning that the agreement of the two ranks is perfect. Position centrality is seen to be strongly correlated to degree and PageRank for some of the data sets, but the two graphs demonstrate that the considered centrality indexes represent different features of the networks.

## A case study about position centrality

To investigate the effect of the parameter *p* on the choice of the center vertices determined by the out-position centrality $P^\text {out}_p(v)$ and the in-position centrality $P^\text {in}_p(v)$ of a vertex *v*, we studied a transportation network for which it is possible to judge by common sense the results of the analysis. Like all transportation networks, it is closely related to the social behaviour of the individuals living in the area of interest.

**Fig. 18:**
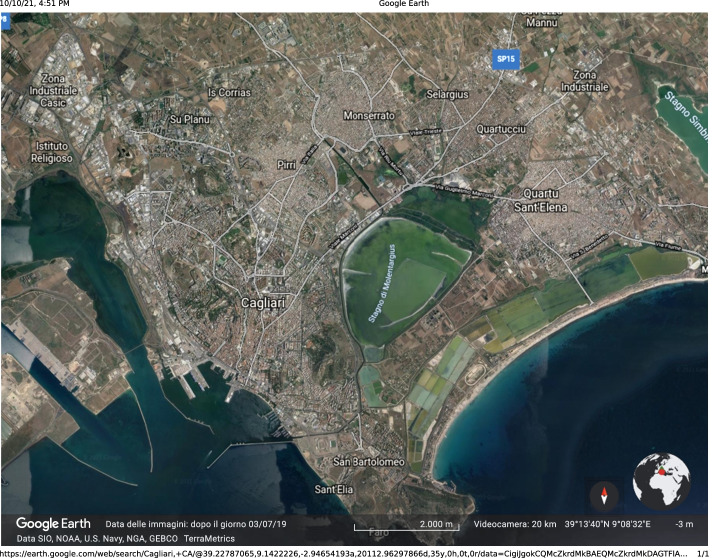
Cagliari metropolitan area; image produced by Google Earth

We considered the bus network that serves the metropolitan area around the town of Cagliari in Sardinia, Italy. The area is about 65 km$^2$, hosts $4.2\cdot 10^5$ people, and includes the town of Cagliari as well as four smaller municipalities very close to Cagliari, contiguous in some parts: Monserrato, Selargius, Quartucciu, and Quartu Sant’Elena; see Fig. 18.

The bus network was constructed using data available on the web. We refer to this network as bus-ca. There are 970 bus stops. They define nodes. The distance between the bus stops is not available. We therefore measure distance as the number of bus stops between the starting and ending nodes on the shortest path. The bus routes define edges. The resulting network is unweighted. Some bus routes depend on the direction of travel, e.g., because some streets are one-way. The bus network therefore is directed.

A bus network, like most geographical networks, is strongly influenced by the landscape and urbanization. The Cagliari commercial center is located on the south-west border of the network, in front of the harbor. A large pond, the Molentargius Saline Regional Nature Park, is located in the center of the urban area. It separates the four municipalities from Cagliari, and prevents straight travel between them.

**Table 3:** Network bus-ca (970 vertices): we report the *p*-out-center and the *p*-in-center vertices for different values of *p*, together with the in/out-centers identified by other centrality measures

*p*	Out-center	$\{\ell ,k\}$	In-center	$\{\ell ,k\}$
$-10$	Zuddas	$\{65,49\}$	Legnano	$\{71,47\}$
$-1$	Zuddas	$\{65,49\}$	Riu Mortu	$\{65,47\}$
0.1	Zuddas	$\{64,49\}$	Riu Mortu	$\{65,47\}$
0.5	San Benedetto	$\{69,55\}$	Giovanni XXIII	$\{66,47\}$
1	San Benedetto	$\{69,55\}$	Roma	$\{68,49\}$
5	Giotto	$\{78,53\}$	Abruzzi	$\{75,49\}$
10	Gherardo delle Notti	$\{79,50\}$	Vergine di Lluc	$\{76,49\}$
Degree	Roma (Sanità)	$\{68,49\}$	Roma (Sanità)	$\{67,49\}$
Betwenness	Brigata Sassari	$\{79,52\}$	Same	
PageRank	Roma (Dogana)	$\{69,49\}$	Same	
HITS	Carlo Felice	$\{67,49\}$	Roma (Sanità)	$\{67,49\}$
H/A centrality	Roma (Dogana)	$\{69,49\}$	Roma (Sanità)	$\{68,49\}$

For each vertex we also report the depth $\ell$ of the associated spanning tree and the minimal lower bandwidth *k* of the corresponding $\{\ell ,k\}$-chained structure

We computed the *p*-out-center and the *p*-in-center vertices of the bus network for different values of *p*. The results are reported in Table 3, where the bus stops are identified by their name. The table also reports the center nodes according to the degree, betweenness (Newman 2010), PageRank (Page et al. 1999), HITS (Kleinberg 1999), and hub/authority centrality (Baglama et al. 2014; Benzi et al. 2013). Since betweenness and PageRank do not distinguish out-centers from in-centers, only one center node is reported for them. For each in/out-center vertex, the depth $\ell$ (i.e., the distance between the center vertex, which is the root, and a most distant leaf of the spanning tree) is reported. Here the distance is measured in terms of the number of edges on the shortest path between the root and the leaf. We also report the minimal lower bandwidth *k* of the $\{\ell ,k\}$-chained graph structure.

**Fig. 19:**
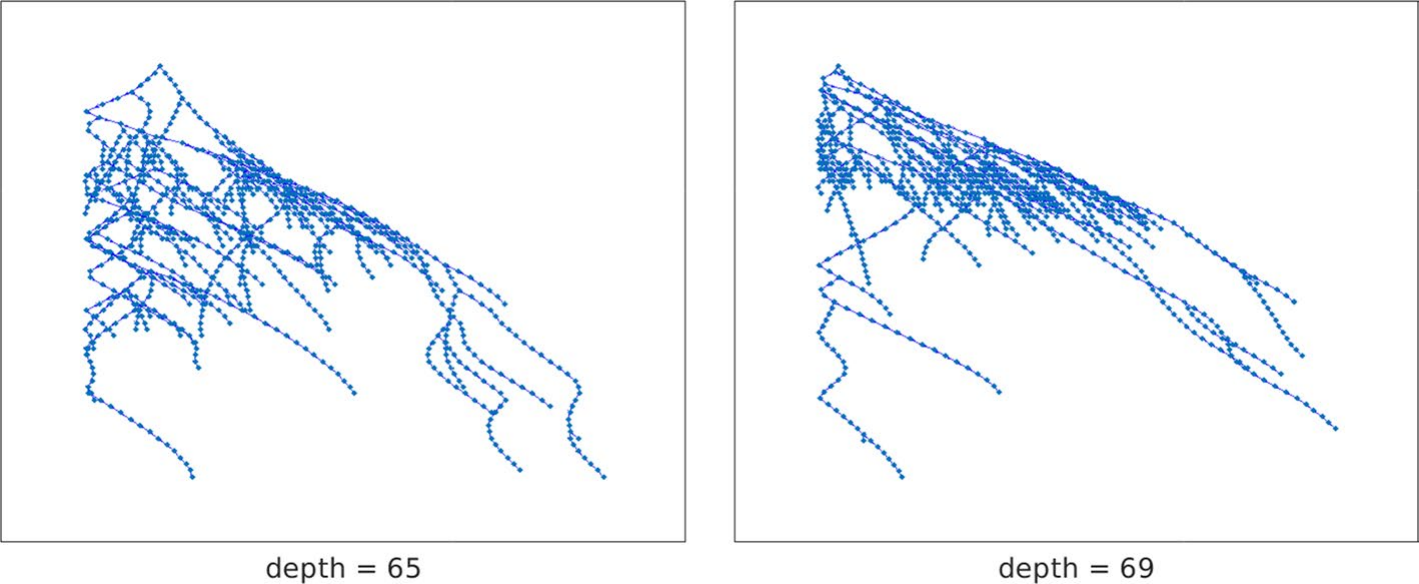
Network bus-ca (970 vertices): on the left, the out-tree spanning the network rooted at the node corresponding to the “Zuddas” bus stop, the out-center vertex for $p=-10,-1,0.1$; on the right, the out-tree rooted at the “San Benedetto” bus stop, out-center node for $p=0.5,1$

The center nodes corresponding to $p=0.5$ and $p=1$ are in the commercial center of Cagliari, the part of town where the largest number of shops and restaurants are located, and where a large number of bus routes converge. The out-tree rooted at the out-center corresponding to the “San Benedetto” bus stop is displayed in the right pane of Fig. 19. The density of nodes in the upper part of the tree shows that many of the first chained sets ${{\mathcal {V}}}_i$, i.e., sets with small index *i*, contain a large number on nodes. This indicates that it is possible to reach a large number of destinations within a small number of bus stops, i.e., in a small time.

When *p* is significantly smaller than one, the out- and in-centralities defined in Definitions 9 and 10 give a smaller weight to sets ${{\mathcal {V}}}_i$ with large cardinality than when *p* is larger than one. The effect is that the spanning tree rooted at the corresponding out-center node is more balanced; see the picture on the left of Fig. 19. This corresponds to a less rapid decay in the number of elements of the sets ${{\mathcal {V}}}_i$ and also to a smaller depth of the tree. The center node “Legnano” is located in the Pirri district of Cagliari, while “Zuddas”, and “Riu Mortu” are in Monserrato, a neighboring municipality. Both these zones join Cagliari with the small towns in the west part of the area, so they are barycentric for the network. It is possible to reach the farthest parts of the network from them after a relatively small number of bus stops.

For a value of *p* somewhat larger than 1, say $p=5$, the center nodes are found in densely populated parts of Cagliari. When the value of *p* becomes very large, the center nodes are suburban bus stops that are served by “strategic” routes that connects them rather easily to the rest of the network.

The depth of the spanning trees, that is, the maximal length of the routes starting from the tree root, increases monotonously with *p*. It is remarkable that the minimal lower bandwidth is rather large. This is a consequence of that there are some bus routes going back towards the center node with only a single bus stop before reaching the center.

The other centrality measures, reported in the lower part of Table 3, with the exception of the betweenness centrality, produce center vertices located in the Cagliari harbor area, the touristic center. In any case, these measures produce results complying with the traditional idea of centrality, while varying the parameter *p* in the position centrality gives the possibility to consider different aspects of this transportation network. The betweenness central vertex is difficult to interpret, as it is located in the central part of the Quartu Sant’Elena town, which does not appear to identify the real center of the network.

## Conclusion

It is important to be able to identify interesting structural properties of directed graphs, because they shed light on how the vertices are connected. This paper introduces the notion of directed chained graphs and illustrates how it helps us to understand the structure of directed graphs. Also, the related notions of in-central and out-central nodes are defined and illustrated. The latter notions are quite intuitive and examples illustrate that they are helpful for identifying important nodes that differ from nodes that are identified by several popular available centrality measures.

## Data Availability

The datasets generated and analyzed during the current study are available from the corresponding author upon request.
